# Anti-tumor efficacy of Selinexor (KPT-330) in gastric cancer is dependent on nuclear accumulation of p53 tumor suppressor

**DOI:** 10.1038/s41598-018-30686-1

**Published:** 2018-08-16

**Authors:** Vinod Vijay Subhash, Mei Shi Yeo, Lingzhi Wang, Shi Hui Tan, Foong Ying Wong, Win Lwin Thuya, Woei Loon Tan, Praveen C. Peethala, Mu Yar Soe, David S. P. Tan, Nisha Padmanabhan, Erkan Baloglu, Sharon Shacham, Patrick Tan, H. Phillip Koeffler, Wei Peng Yong

**Affiliations:** 1 Cancer Science Institute of Singapore, Yong loo Lin School of Medicine, National University of Singapore, Singapore, Singapore; 2 MRC Cancer Unit, University of Cambridge, Hutchison/MRC Research Centre, Cambridge, United Kingdom; 3 Department of Haematology-Oncology, National University Hospital, Singapore, Singapore; 4 Department of Cancer and Stem Cell Biology, Duke-NUS Graduate Medical School, Singapore, Singapore; 5 Karyopharm Therapeutics Inc, Newton, Massachusetts, USA

## Abstract

Exportin-1 (XPO1) controls the nucleo-cytoplasmic trafficking of several key growth regulatory and tumor suppressor proteins. Nuclear export blockade through XPO1 inhibition is a target for therapeutic inhibition in many cancers. Studies have suggested XPO1 upregulation as an indicator of poor prognosis in gastric cancer. In the current study, we investigated the anti-tumor efficacy of selective inhibitors of nuclear export (SINE) compounds KPT-185, KTP-276 and clinical stage selinexor (KPT-330) in gastric cancer. XPO1 was found to be overexpressed in gastric cancer as compared to adjacent normal tissues and was correlated with poor survival outcomes. Among the 3 SINE compounds, *in vitro* targeting of XPO1 with selinexor resulted in greatest potency with significant anti-proliferative effects at nano molar concentrations. XPO1 inhibition by selinexor resulted in nuclear accumulation of p53, causing cell cycle arrest and apoptosis. Also, inhibition of XPO1 lead to the cytoplasmic retention of p21 and suppression of survivin. Orally administered selienxor caused significant inhibition of tumor growth in xenograft models of gastric cancer. Furthermore, combination of selinexor with irinotecan exhibited greater anti-tumor effect compared to individual treatment. Taken together, our study underscores the therapeutic utility of XPO1 targeting in gastric cancer and suggests the potential benefits of XPO1 inhibition in-combination with chemotherapy.

## Introduction

Intracellular location of tumor suppressor proteins (TSPs) and growth regulatory proteins (GRPs) is critical to cancer cells for proliferation and survival^1^. Many of these proteins must localize to the cell nucleus to prevent cancer initiation, progression and resistance to chemotherapy. During malignant transformation or in response to the tumor environment, cancer cells appear to acquire intracellular mechanisms for nuclear exclusion of tumor suppressor proteins^2^. In this regard, therapeutic targeting of the nucleo-cytoplasmic shuttling of macromolecules has emerged as a promising approach in cancer treatment^3^. Exportin1 (XPO1) also called chromosome region maintenance 1 (CRM1), is a key nuclear export protein that mediates protein export from the nucleus to the cytoplasm through the nuclear pore complex. It is a member of the karyopherin β protein family of transport receptors that can recognize cargo proteins with leucine-rich nuclear export signals (NESs)^4^. XPO1 is the primary nuclear exporter of a number of tumor suppressor and cell cycle regulatory proteins such as p53, p21, FOXO1, cyclin B1/D1 and chemotherapeutic targets such as DNA topoisomerases I and II alpha^5,6^. Overexpression of XPO1 was shown to be associated with poor prognosis or resistance to chemotherapy in various cancers^7^. XPO1 upregulation also results in enhanced nuclear-cytoplasmic transport. This causes mislocalization, inactivation or aberrant activation of tumor suppressor proteins (TSPs), thereby resulting in oncogenesis^8^. In gastric cancer, high expression of XPO1 was shown to have a significant correlation with advanced tumor stage, distant metastasis and poor prognosis^9^.

Leptomycin B was the first XPO1 inhibitor shown to efficiently inhibit nuclear export in various cancer cell lines *in vitro*^10^. However, its clinical development was subsequently limited due to severe toxicities without significant efficacy in human phase I trial^11^. Selective inhibitor of nuclear export (SINE) compounds (KPT-185, KPT-276, and KPT-330) were developed based on the fact that Leptomycin B specifically binds to the Cys528 residue in the cargo-binding groove of XPO1 to restore the function of TSPs in the nucleus, leading them to induce cancer-specific apoptosis^7^. SINE compounds have shown anti-tumor activity in several solid and hematological malignancies in preclinical *in vitro* and/or *in vivo* studies, including renal cancer^12^, pancreatic cancer^13^, prostate cancer^14^, breast cancer^15^, melanoma^16^, multiple myeloma^17^, mantle cell lymphoma^18^, chronic lymphocytic leukemia^7^, acute myeloid leukemia^19,20^ and non-Hodgkin lymphoma^21^. Further to the Phase1 clinical trials of oral KPT-330 (selinexor, Karyopharm Therapeutics) for both solid tumors (NCT01607905, NCT01896505) and advanced hematologic malignancies (NCT01607892), the compound is currently undergoing Phase 2/3 trials. In addition, given the noted synergistic effects on cytotoxicity in neoplastic cells, the potential for combining XPO1 inhibition with conventional chemotherapy, such as topoisomerase inhibitors, is also being explored in recent clinical trials (NCT02283359). A recently concluded phase II trial that tested selinexor in combination with dexamethasone showed an overall response rate (ORR) of 21% in patients with heavily pretreated, refractory myeloma^22^. In the present study, we investigated the therapeutic efficiency of XPO1 inhibition with SINE compounds KPT-185, KPT-276 and KPT-330 in gastric cancer. The mechanism of XPO1 inhibitor-mediated proliferation inhibition and apoptosis in gastric cancer cells is also determined. Furthermore, the anti-tumor efficacy of KPT-330 is also characterized using a xenograft model of gastric cancer.

## Methods

### Patient tissue samples and genomic profiling

An independent patient cohort including 153 tumors and 100 normal samples were profiled for gene expression using Affymetrix human genome U133 Plus 2.0 GeneChips (Affymetrix, Santa Clara, CA). Gene expression microarray data is available under GEO accession number GSE15460. All primary gastric tissues were obtained from the National University Hospital, Singapore or National Cancer Centre, Singapore tissue repositories with approvals from the Research Ethics Review Committee, National University Hospital and Institutional Review Board, National Cancer Centre. All methods were performed in accordance with the relevant guidelines and regulations and signed patient informed consent. Tumor samples were histologically confirmed to contain cancer cells, with an average tumor cellularity of 40%. Non-malignant samples (normal tissue) were harvested from stomach tissue distant from the tumor and exhibiting no visible evidence of tumor or intestinal metaplasia/dysplasia upon surgical assessment. Histopathological data and patient characteristics of the cohort is provided in a previous publication^23^.

### Cell lines and reagents

Gastric cancer cell lines, AGS and NCI-N87 were purchased from ATCC, USA. IM95, NUGC-3 and NUGC-4 were purchased from Health Science Research Resources Bank, Japan. MKN1, MKN45, TMK1 and YCC10 were obtained from DUKE NUS, Singapore. HGC-27 was purchased from Public Health England, UK. All cell lines except YCC10 cell lines were cultured in RPMI 1640 medium (Gibco; Grand Island, NY) containing 10% heat inactivated fetal bovine serum (Gibco; Grand Island, NY) and 1% penicillin/streptomycin (Gibco; Grand Island, NY). YCC10 cell lines was cultured in MEM supplemented with 20% heat inactivated fetal bovine serum (Gibco; Grand Island, NY), 1% penicillin/streptomycin (Gibco; Grand Island, NY) and 1% sodium pyruvate (Gibco; Grand Island, NY). All cell lines are maintained at 37 °C in humidified atmosphere containing 5% CO_2_.

### Reagents, antibodies and materials

KPT-185, KPT-276 and KPT-330 were provided by Karyopharm Therapeutics and dissolved in DMSO for *in vitro* studies. KPT-330 was diluted in 0.6% Pluronic® F68 and PVP K-29/32 solution for the *in vivo* study. Irinotecan was purchased from Pfizer (Pfizer; NY, USA) and stored at 4 °C. Primary antibodies including anti-caspase-3, anti-caspase-9, anti-p53, anti-p21, anti-survivin, anti-β-tubulin, anti-lamin, anti-p27, GAPDH were purchased from Cell Signaling Technology (Cell Signaling; MA, USA). Primary anti-XPO1 was purchased from Proteintech (Proteintech; IL, USA). Secondary antibodies (anti-rabbit IgG, HRP-Linked and anti-mouse IgG, HRP-Linked) were purchased from Cell Signaling Technology (Cell Signaling; MA, USA). Alexa Fluor® 568 Goat Anti-Rabbit IgG and ProLong® Gold Antifade Reagent with DAPI were purchased from Molecular Probes (Life Technologies; CA, USA).

### Quantitative PCR (qPCR) analysis

Total RNA was extracted from cultured cells with RNeasy Mini kit (Qiagen, CA, USA) with the use of QIAshredder spin column for homogenization and an on-column DNase digestion. 2 µg of the total RNA was reversely transcribed using M-MLV reverse transcriptase enzyme (Promega, WI, USA). The cDNA obtained was analysed quantitatively using Power SYBR Green PCR Master Mix (Applied Biosystems, CA, USA) on an ABI7300 Real-time PCR system. Primers used are listed in Table 1. Cycling conditions were 95 °C for 15 min, 40 cycles of 15 s at 94 °C, 30 s at 55 °C and 30 s at 72 °C. Ct values were generated using default analysis settings. Relative quantification (RQ) was calculated using 2^−ΔΔCT^ method.

**Table 1:** Univariate and Multivariate analysis of the correlation between clinicopathological parameters and overall survival of patients with GC.

	N = 153	**OS**		
		**Univariate**	**Multivariate**	
		P value (Chi square)	Adjusted P value	HR(95%CI)
**Gender**				
Male	99	0.017	0.169	
Female	54			
**Gene expression**				
High (≥4281.84)	77	0.037	0.021*	1.641(1.079–2.496)
Low (<4281.84)	76			
**T stage**				
T1-T2	24	0.000	0.164	
T3-T4	127			
Not reported	2			
**N stage**				
N0-N1	69	0.000	0.815	
N2-N3	82			
Not reported	2			
**M stage**				
M0	122	0.000	0.765	
M1	30			
Not reported	1			
**Stage (AJCC7)**				
Stage1&Stage 2	44	0.000	0.001*	0.264(0.119–0.588)
Stage 3&Stage 4	108			
Not reported	1			
**Histo Grade**				
Well& Mod differentiated	54	0.367		
Poorly& Undifferentiated	99			
**Lauren**				
Diffuse	62	0.469		
Non-diffuse	91			

### Cell viability and proliferation assays

To assess the chemo sensitivity of tumor cells to SINE compounds, cell viability was measured by CellTiter 96® Aqueous Non-Radioactive Cell Proliferation Assay (Promega; WI, USA). Cell suspension was cultured in 96-well flat-bottomed micro titer plates at seeding density of 2 × 10^3^ cells/well and incubated overnight. Drugs were tested at concentrations ranging from 0.1 µM to 10.0 µM. Micro titer wells containing tumour cells without drug treatment served as controls, and wells containing complete medium served as blank controls. Each drug was tested in triplicate. Cells were incubated for 72 hours before the addition of the assay reagent (1 mg/mL per well) and absorbance was read at 550 nm using a spectrophotometric micro plate reader (Tecan; Männedorf, Switzerland). The percentage cell viability to different drug concentrations was calculated as the inhibition rate of (mean absorbance of treated wells/mean absorbance of control wells) × 100%. IC_50_ was calculated by GraphPad Prism v4.0 (GraphPad Software, Inc; CA, USA).

### Cell cycle analysis

Cell cycle distribution of cells was analyzed using flow cytometry. Cells were fixed with ice-cold 70% ethanol at 4 °C overnight, and washed in PBS. Cells were then resuspended in PBS RNase (Qiagen, CA, USA) and propidium iodide (BD Pharmingen™, CA, USA) and incubated for 15 min at room temperature. Cell cycle analysis was performed on a BD LSRII Analyzer equipped with FlowJo software (version vX 0.7). A total of 10,000 cells were analyzed for each sample.

### Apoptosis assay

Apoptosis was detected by Annexin V-FITC (fluorescein isothiocyanate) kit (BD Pharmingen) according to manufacturer’s instructions. Briefly, the cells (1 × 10^5^ cells/ml) were grown to 80% confluency in 25 cm^2^ flasks in F12K supplemented with 10% fetal calf serum. After 24 hours of drug treatment, cells were harvested, washed thrice with cold PBS and resuspended in 1x binding buffer. An aliquot of 100 µl of the cell suspension was transferred into a microfuge tube and mixed with equal volumes (5 µl) of Annexin V- FITC and Propidium Iodide (PI). The cells were gently vortexed and incubated for 15 minutes at 37 °C in the dark, before the addition of 400 µl of 1X binding buffer in each tube. The cell samples were then analyzed using a flow cytometer BD^TM^ LSR II (BD biosciences, CA, USA) equipped with FlowJo software (version vX 0.7).

### Western blot and protein analysis

Cells treated with SINE compounds were washed with ice cold PBS and resuspended in lysis buffer (CelLytic; Sigma-Aldrich; St Louis, MO) containing protease inhibitor cocktail (Roche; Mannheim, Germany). Lysates were sonicated, incubated on ice for 20 min and centrifuged at 14000 rpm for 20 min at 4 °C. Protein concentrations were determined using Bradford assay (Bio-Rad, CA, USA). Nuclear and cytoplasmic extracts were prepared with NE-PER Kit (Pierce; IL, USA). 20 µg of protein samples were electrophoretically separated on 12% SDS-PAGE and electro-transferred to a PVDF membrane (Immun-Blot PVDF; Bio-Rad, CA, USA). Membranes were blocked for an hour at room temperature in 5% non-fat dry milk (Bio-Rad; CA, USA) and subsequently incubated with primary antibodies overnight at 4 °C. After three washes with Tween 20 in PBS, membranes were incubated for 1 h at room temperature with corresponding horseradish peroxidase conjugated anti-rabbit and anti-mouse secondary antibodies. The membranes were washed four times with PBS/Tween 20, and the signals were visualized by ECL reagent (AmershamTM ECL Plus Western Blotting Detection System; GE Healthcare; Buckinghamshire, UK), followed by exposure to chemiluminescence film (Amersham Hyperfilm^TM^ ECL; GE Healthcare; Buckinghamshire, UK). Immunoblot analyses were repeated at least twice for each protein tested.

### Immunofluorescence Staining

Cells were seeded onto the 8 wells chamber slide and allowed to grow until 70% confluence. The cells were fixed with 4% formaldehyde for 10 minutes and permeabilised by 0.1% Triton X100 in PBS (PBST) for 10 minutes. The slides were blocked with 1% Bovine Serum Albumin (BSA) in PBST for an hour at room temperature. The cells were incubated overnight with primary antibody p53 and p21 (Cell Signaling, MA, USA) at 4 °C and subsequently incubated with secondary antibody conjugated with Alexa Fluor 568 for an hour in the dark at room temperature. Prolong Gold with DAPI was added into each wells and air-dried in the dark to detect nuclei. The slides were sealed with coverslips for imaging using a confocal laser microscope (Olympus FluoView^TM^ FV1000).

### GC xenograft murine model

4–6 week old NOD-SCID mice (n = 50) were injected subcutaneously with AGS gastric cell lines (5 × 10^6^) suspended in Matrigel (BD biosciences). Six animals were randomly assigned to each treatment group (1) Vehicle (0.6% pluronic F-68 and 0.6% Plasdone K-29/32) (2) Irinotecan (20mpk, i.v) (3) KPT-330 (10mpk, oral-gavage) (4) combination of irinotecan and KPT-330 (intra venous/ oral-gavage). Treatment was initiated when the tumors reached an average volume of 100 mm^3^ and continued for 2 weeks. Tumor growth was followed by bi-weekly measurements of tumor diameters with a Vernier caliper and tumor volume (TV) was calculated according to the formula: TV = (W^2^ X L)/2 mm^3^, where ‘W’ is the width and ‘L’ is the length. The anti-tumor activity was assessed as TV inhibition percentage (TVI%) in treated versus control mice, calculated as follows: TVI% = 100-(mean TV treated/mean TV control x100). The experiments were performed in accordance with Institutional Animal Care and Use Committee (IACUC) guidelines, National University of Singapore and the experimental protocol was approved by IACUC under the protocol number 0469-R1. Mice were sacrificed if they had a tumor greater than 1.5 cm in diameter, if total tumor burden was greater than 10% of body weight, or if a tumor ulcerated or interfered with mobility

### Statistical analysis

Statistical analysis was performed by using SPSS 11.0 statistical software program (SPSS, Chicago, IL, USA). Differential analyses between tumor and normal samples were evaluated using Student’s t-test, and association between gene expression and clinopathological features was assessed using Pearson correlation. Survival curves were plotted using Kaplan-Meier and statistical significance was assessed using the Cox proportional hazards model. Note: XPO1 high and low expressed groups were determined using the median of respective distributions. These analyses were performed using the statistical package in R (www.r-project.org). A *p*-value of less than 0.05 was considered statistically significant.

## Results

### XPO1 upregulation and its association with overall survival in GC patients

**Figure 1:**
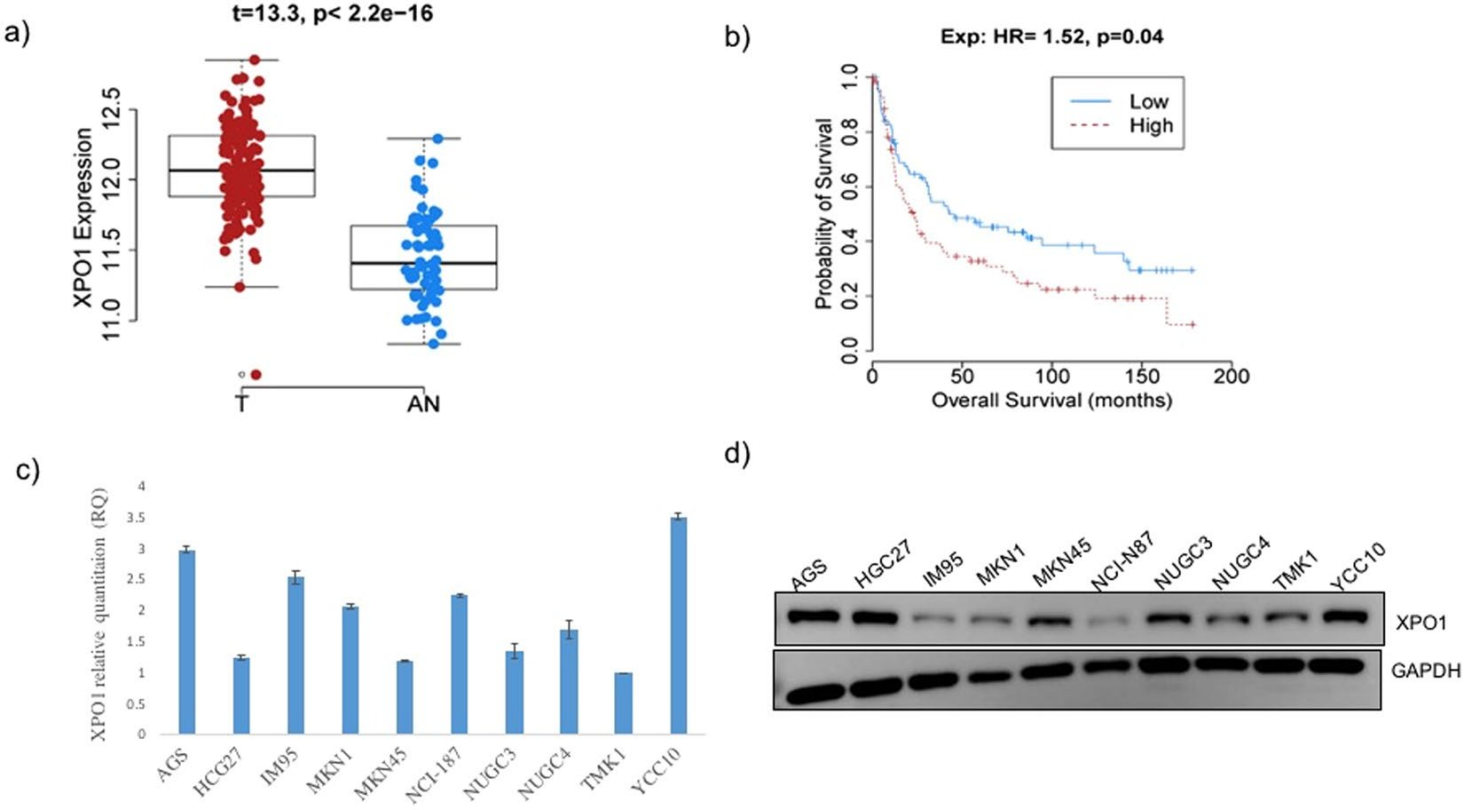
(**a**) XPO1 overexpression in gastric tumor tissues (red, n = 200) as compared to normal gastric tissues (blue, n = 100) (t = 13.3, p < 2.2e-16). (**b**) Survival analysis of gastric cancer patients with XPO1 over expression. (**c**) XPO1 gene expression in gastric cancer cells detected by q–PCR. Relative gene expression is represented as averaged relative quantity in the Y-axis. (**d**) XPO1 gene expression in gastric cancer cells detected by immunoblot analysis. GAPDH serve as internal control.

Microarray analysis of clinical samples revealed that XPO1 is overexpressed in gastric cancer as compared to adjacent normal tissues (Fig. 1a). Based on the median expression value, 77 tumors were classified as high whereas the remaining 76 were classified as low in XPO1 expression. None of the tumors showed an absence of XPO1 expression. Interestingly, high XPO1 expression was found to be associated with poor survival outcomes of patients (Fig. 1b). Furthermore, univariate and multivariate analysis of clinicopathological parameters suggested AJCC stage and XPO1 expression as the only independent prognostic factors (Table 1). Expression levels of XPO1 was also determined in a panel of 10 gastric cancer cell lines. All cell lines showed detectable expression levels of XPO1, among which AGS and YCC10 showed the highest expression (Fig. 1c,d).

### Effects of XPO1 inhibition on proliferation in GC cell lines

**Figure 2:**
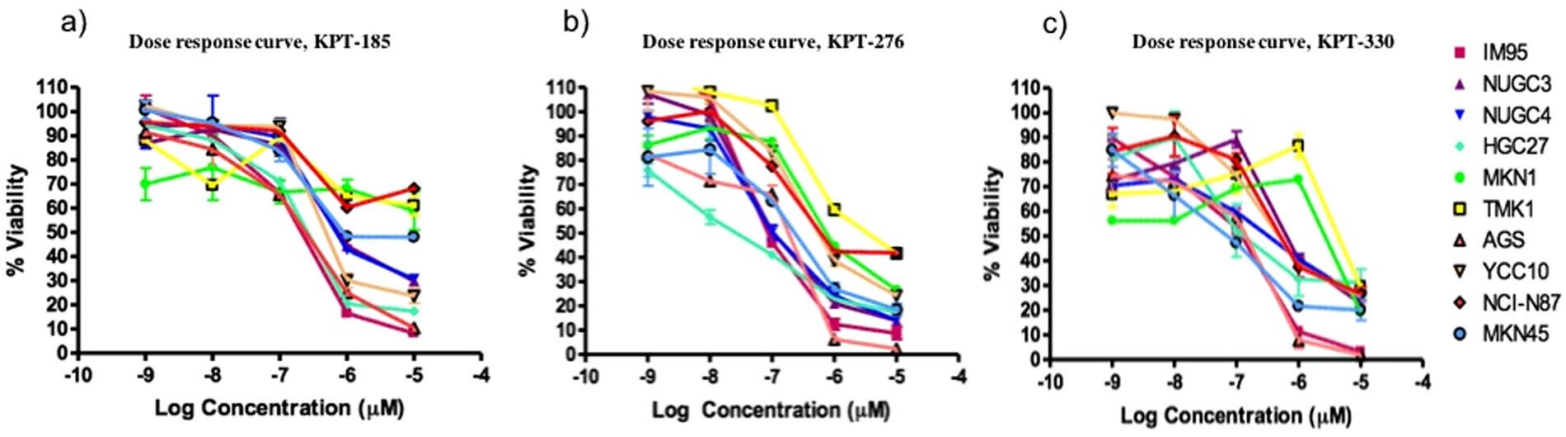
IC_50_ of KPT-SINE analogs to 10 gastric cancer cell lines. Mean IC_50_ ± Standard Error (SE) of at least two independent experiments were performed in triplicates. Cells were treated with KPT-SINE analogs for 72 h and cell proliferation was assessed using the MTS assay.

To evaluate the growth-inhibitory effect of KPT-SINE analogs, IC_50_ concentration of KPT-185, KPT-276 and KPT-330 were determined in gastric cancer cells (Fig. 2a–c). Out of the three SINE analogs, the cell lines were most sensitive to XPO1 inhibition by KPT-330 that resulted in a significant inhibition of cell proliferation with IC_50_ values ranging from 36 nM to 1.6 µM. KPT-185 also caused marked inhibition in cell proliferation, however appeared less potent than KPT-330 with IC_50_ values in the range of 68 nM to 4.2 µM. KPT-276 has the least potent effect on growth of GC cell lines whereby 3 of the 10 cell lines were found to be resistant with IC_50_ over 10 µM.

### Apoptosis induction and cell cycle effects in GC cells treated with KPT-330

**Figure 3:**
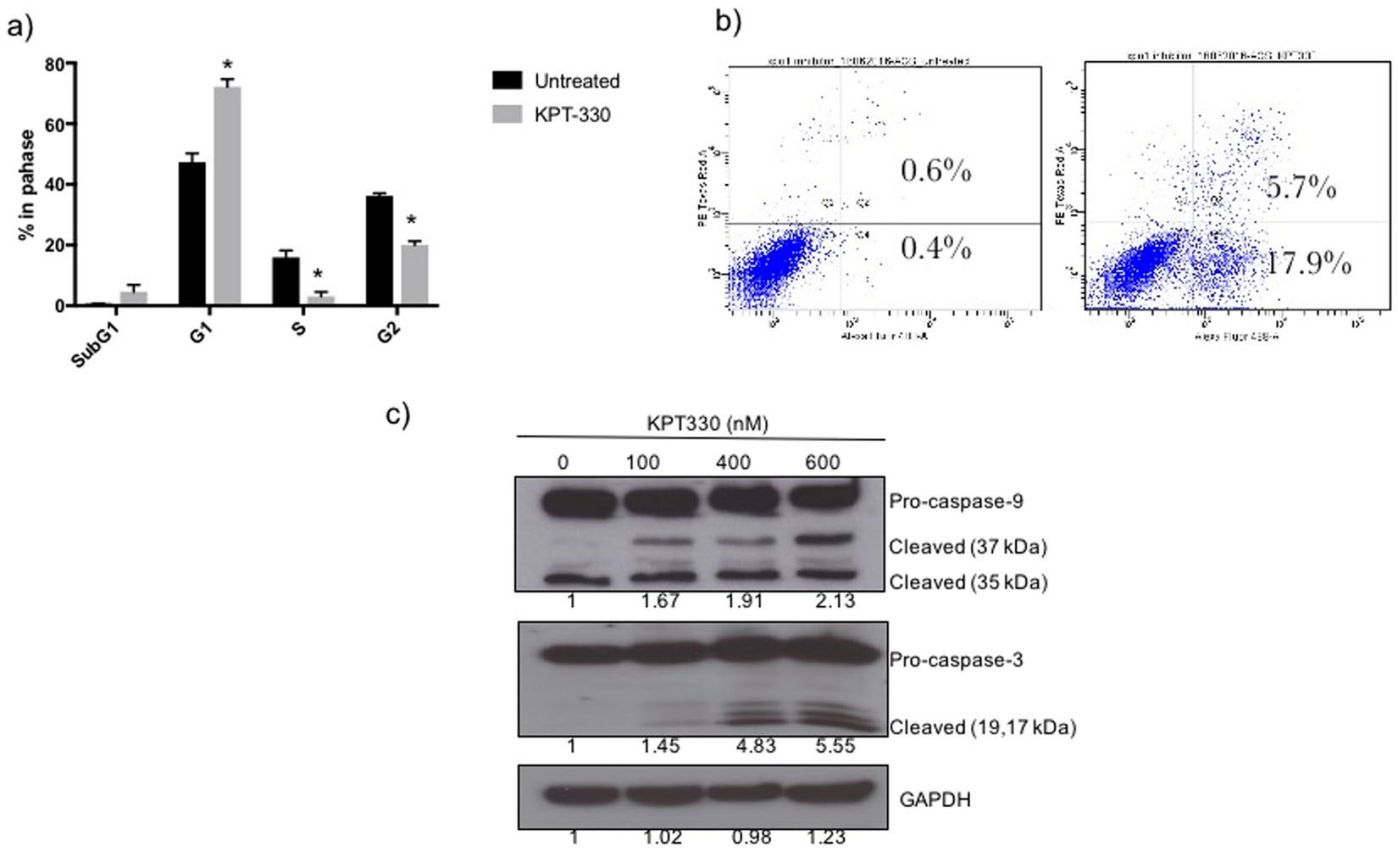
AGS cells treated with IC50 concentrations of KPT-330 for 24 hours was analysed for cell cycle profiles and apoptosis (**a**) Cell cycle analysis (**b**) Annexin-FITC apoptosis assay. (**c**) Immunoblots of apoptotic protein expressions in AGS cells treated with varying doses of KPT-330 for 24 hours. The values reported under each blot indicates the percentage expression of cleaved caspase 3 & 9 relative to controls taken as 1 after normalization by GAPDH (Image J quantification). The results shown are representative of two independent experiments.*p-value of less than 0.05 was considered statistically significant.

The effect of XPO1 inhibition in cell cycle regulation and apoptosis was determined in AGS cells that showed high XPO1 expression and KPT-330 sensitivity. Treatment of AGS cells with KPT-330 resulted in cell cycle arrest at G1 phase, concomitant with an increase in sub-G1 population. On the other hand, a significant decrease in the S and G2 phase were also observed in the treated cells as compared to the untreated ones (Fig. 3a, P value < 0.05). Moreover, KPT-330 treatment also resulted in a significant increase in apoptotic population (23.6%) with 17.9% cells entering early apoptosis as compared to untreated cells. (Fig. 3b, P value < 0.05).

The effect of KPT-330 in cell cycle regulation and apoptotic induction was determined in YCC10 cells to confirm that the observation is consistent across multiple cell lines. Both AGS and YCC10 showed significant increase in KPT-330 induced cellular apoptosis as compared to untreated cells (Supplementary Fig. S2). To further confirm apoptosis induction in KPT-330 treated cells, we examined the cleavage of caspase-3 and caspase-9 in drug treated cells. As shown in Fig. 3c, AGS cells treated with KPT-330 showed a dose dependent increase in expression of cleaved caspase-3 and caspase-9 (active form). The expression of cleaved forms of caspase-3 & 9 was detectable at dosing concentrations as low as 100 nM, hence suggesting a caspase dependent induction of apoptosis in KPT-330 treated cells.

### KPT-SINE induced apoptosis in GC cells was mediated by p53 and CRM1

**Figure 4:**
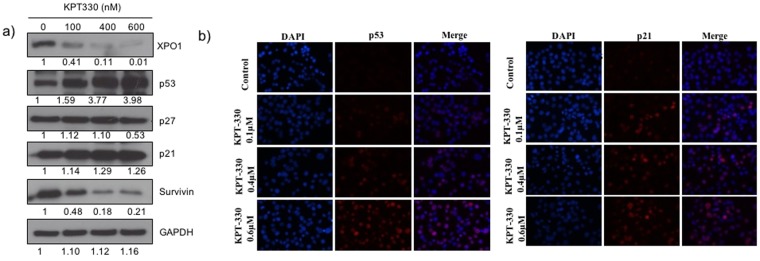
(**a**) Immunoblots of tumor suppressor protein expression in AGS cells treated with KPT-330 for 24 hours. The values reported under each blot indicates the percentage expression relative to controls taken as one after normalization by GAPDH (Image J quantification). The results shown are representative of two independent experiments. (**b**) Immunofluorescence of p53 and p21 expression in KPT-330 treated AGS cells. P53 and p21 stained with Alexa Flour568 (red) and cell nuclei stained with DAPI (Blue).

To further investigate the mechanism of apoptosis induced by the KPT-330, we examined the expression profiles of apoptotic proteins in drug treated AGS cells (Fig. 4a). Expectedly, treatment of cells with KPT-330 lead to a dose-dependent decrease in XPO1 expression levels. A resultant effect of this was observed with marked upregulation of p53 and its transcriptional target p21. On the other hand, a decrease in expression of anti-apoptotic survivin was also observed hence suggesting a KPT-330 triggered induction of cell death. The subcellular localization of p53 and p21 was determined by immunofluorescence analysis. As shown in Fig. (4b), treatment with KPT-330 resulted in a dose-dependent upregulation of p53 and p21 expression in cell nuclei, hence suggesting a gain in apoptotic function.

**Figure 5:**
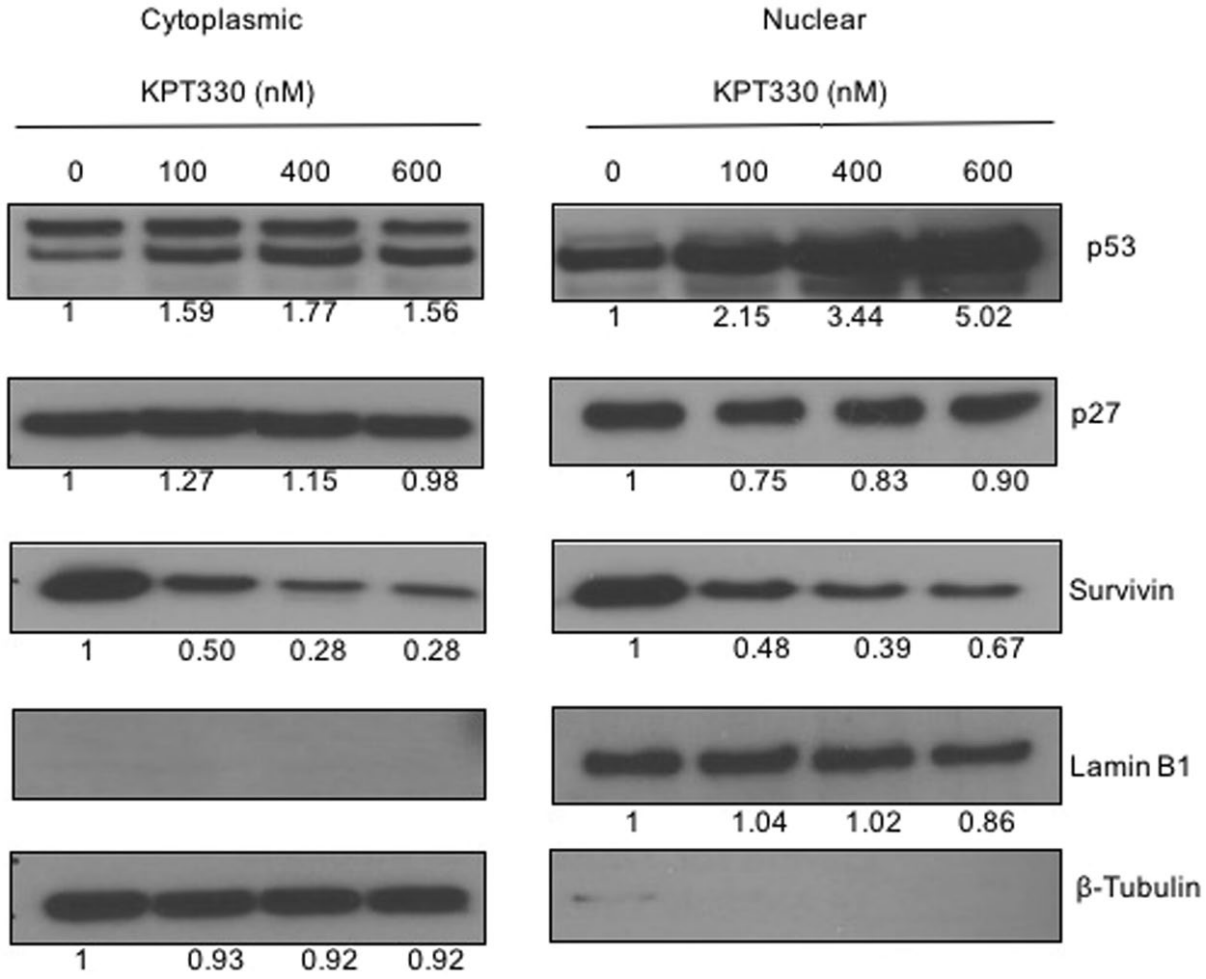
Immunoblot of protein expression in cytoplasmic and nuclear fractions of AGS cells treated with KPT-330. Lamin B1 and β- tubulin serve as loading controls for nuclear and cytoplasmic fractions, respectively. The values reported under each blot indicates the percentage expression relative to controls taken as 1 after normalization by GAPDH (Image J quantification). The results shown are representative of at least two independent experiments.

The KPT-330 induced effects in the intracellular localization of other oncogenic determinants were also evaluated. The cytoplasmic and nuclear fractions of KPT-330 treated AGS cells were extracted and subjected to immunoblot analysis (Fig. 5). Consistently, KPT-330 led to the nuclear retention and upregulation of p53 expression in AGS cells. In contrary, the expression of p27, a non p53 regulated protein, remained unchanged in the nucleus, whereas an increase in its cytoplasmic expression was observed. Interestingly, a decrease in expression of survivin was observed both in the cytoplasmic and nuclear fractions of KPT-330 treated cells.

### KPT-330 inhibits growth of gastric cancer cells in a xenograft model

**Figure 6:**
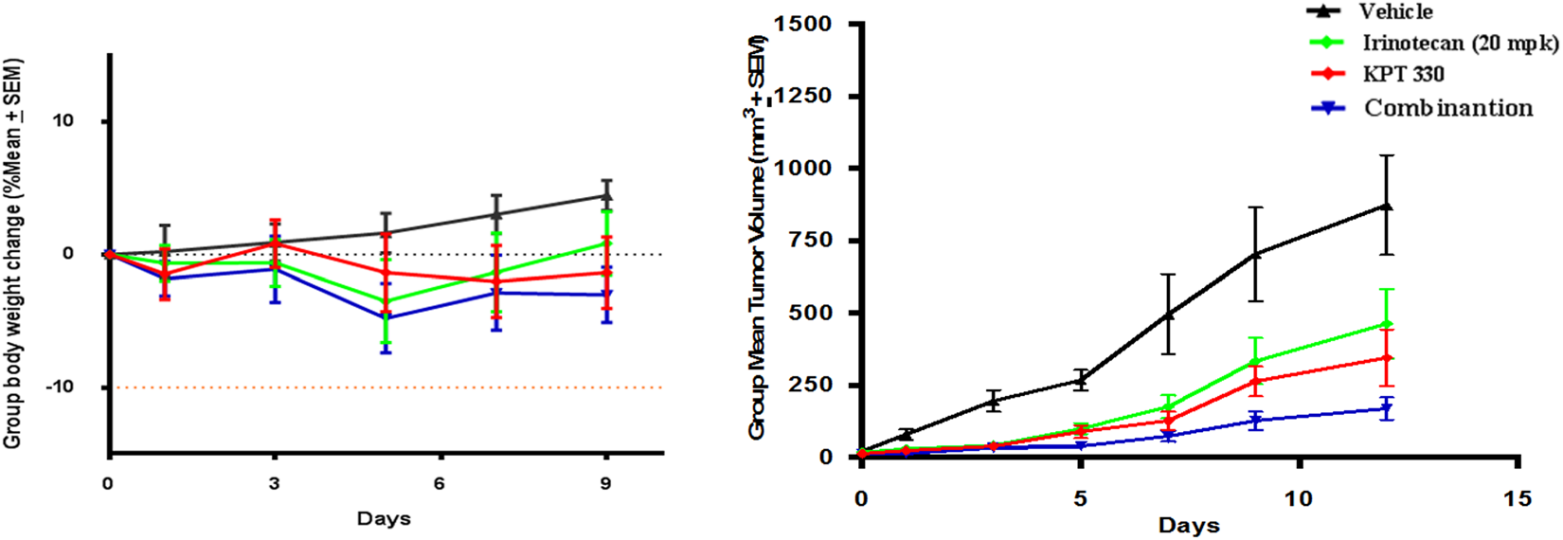
NOD-SCID mice were injected subcutaneously with AGS cells. Six animals were randomly assigned to each group and treated for 2 weeks. (**a**) Evaluation of body weight (**b**) Mean tumour volume (mm^3^).

The effect of XPO1 inhibition in tumor growth was determined *in vivo* using a mouse xenograft model. NOD-SCID mice transplanted with AGS cells showed a remarkable decease in tumor growth after treatment with KPT-330 as compared to the mice treated with vehicle controls. Since preclinical evaluation of XPO1 inhibition in combination with chemotherapy yielded promising results in other cancers^24,25^, we evaluated the combinatorial effect of KPT-330 with irinotecan in gastric cancer (Fig. 6). The xenografts showed significant anti-tumor effects can be achieved by irinotecan monotherapy. Strikingly, a synergistic effect of KPT-330 was observed in combination of KPT-330 with irinotecan, causing greater appreciable anti-tumor effects. The SINE compound was well tolerated with no toxic deaths and minimal weight loss. In addition, no gross pathology was observed at necropsy carried out at the end of the experiment. Consistent with the *in vitro* data, KPT-330 treatment induced an upregulation of P53 and p21 expression in *in vivo* xenografts tissues. An increase in p21 expression was also seen in irinotecan and combinatorial treatment samples, while p53 showed a modest increase as compared to vehicle controls. Furthermore, immunohistochemical analysis revealed a decrease in ki67 expression in all treated samples as against the vehicle controls (Supplementary Fig. S3).

## Discussion

The requirement for nucleo-cytoplasmic export of key proteins in normal cells is suggested to be less stringent that that in malignant cells^19^. This “Achilles heel” of malignant cells could be targeted using nuclear export inhibitors to achieve therapeutic benefits. In this regard, selective killing of tumor cells could be achieved through restoration and reactivation of tumor suppressors. XPO1 is highly expressed in many tumor types and coordinates the nucleo-cytoplasmic export of ~220 proteins. This includes several tumor suppressors and mediators of proliferative and pro-survival signaling pathways^26,27^. The current study identified XPO1 as an independent prognosis indicator, as its over expression correlates with poor survival in gastric cancer patients. XPO1 expression was shown to be significantly high in diffuse type of GC, however no association was observed between XPO1 expression with stage and grade of cancer (Supplementary Table S1). The three SINE compounds (KPT-185, KPT-276 and KPT-330) presented in our study were previously shown to induce covalent modification of the Cys528 residue at the NES groove of XPO1 protein^7^. They are structurally similar, but differ in the pharmacokinetic properties. KPT-185 was shown to have poor PK properties, hence making it unsuitable for *in vivo* study. On the other hand, KPT-276 and KPT-330 exhibit optimal PK properties^20,25^. Here, we establish the anti-tumor efficacy of XPO1 inhibitor KPT-330, both in *in vitro* and *in vivo* models of gastric cancer. Our findings fall in-line with the previous reports that suggested anti-tumor efficacy of XPO1-targeting drugs in various hematological malignancies and solid tumors^28,29^.

Since XPO1 inhibition results in nuclear retention and activation of multiple TSPs, its anti-tumor effects could largely be independent of underlying oncogenic drivers that are key in maintaining a neoplastic state^26^. Although the exact mechanism underling the selective anti-tumor efficacy of KPT-330 remain uncharacterized, a major mediatory role of p53 is assumed. As a nuclear export mediator, XPO1 channels the nuclear export of p53. This leads to the cytoplasmic degradation and functional inactivation of p53 as a tumor suppressor^2,30^. XPO1 inhibition by KPT-330 abrogates p53 degradation through its nuclear retention. When p53 is activated in the nucleus, it could promote either cell cycle arrest or apoptotic cell death^31^. In our study, the activation of caspase-3 and 9 in KPT-330 treated cells is a clear indication of induction of apoptosis. Other proteins that were found to be affected by XPO1 inhibition include tumor suppressor protein p21 and survivin. The upregulation of p21, a transcription target of p53, is suggestive of the restoration/reactivation of p53 tumor suppressor function. Of note, the molecular function of p21 depends on its cellular localization as it plays different roles in nucleus and in cytoplasm. Nuclear p21 induces cell cycle arrest in response to DNA damage, whereas its cytoplasmic expression corresponds to an anti-apoptotic role.^32^. As shown by immunofluorescence microscopy, p21 expression was retained in the nucleus upon CRM1 inhibition (Fig. 5). Our finding that XPO1 inhibition restored p53 nuclear function, causing cell cycle arrest and apoptosis is consistent with the earlier studies^27,33^. No changes in expression of p27, a non-p53-regulated cell cycle inhibitor, was observed. Intriguingly, XPO1 inhibition by KPT-330 also lead to a decrease in survivin expression levels. Survivin constantly shuttles between the nucleus and the cytoplasm and plays a major role in apoptosis regulation and control of cell cycle progression at the G2/M phase^34^. Elevated level of survivin has been reported to promote tumorogenesis and confer chemotherapy resistance^35,36^. Our study adds further credence to previous reports that suggested strategies aimed at down-regulating survivin as a therapeutic approach^37^.

The combination of molecularly targeted agents with chemotherapy has shown the potential of improving the outcomes of cancer patients. In earlier studies, the ability of XPO1 inhibitors to induce apoptosis or anti-proliferative effects in cell lines was shown to have improved synergistically when combined with chemotherapeutic drugs^38^. Moreover, XPO1 inhibition was shown to induce sequestration of topoisomaerase 1 (TOP1) in the nucleus^39^. This would sensitize the cells to TOP1 inhibitors and the effect could be further enhanced with combinational therapeutic regimens. Here, we also evaluated the anti-tumor efficacy of KPT-330 in combination with irinotecan in xenograft models of gastric cancer. Irinotecan, a topoisomerase 1 inhibitor, is a commonly used chemotherapeutic agent in gastric cancer. It binds to Top I-DNA complex and prevents re-ligation of double stranded DNA during DNA replication^40^. Interestingly, combination of KPT-330 with irinotecan resulted in a remarkable decrease of tumor growth, *in-vivo*. This synergy observed between KPT-330 and irinotecan generates further interest since combination regimens that accelerate time to remission and overcome resistance is pivotal in cancer therapy.

The present study strongly correlates XPO1 expression with poor prognosis in gastric cancer. Future studies should explore if XPO1 inhibition induces a shift in balance of neoplastic cells from a hyper proliferative state to an apoptotic state that could further be enhanced by concomitant targeting of other crucial cellular processes. The continued evaluation of KPT-330 to establish the safety of targeting nuclear export through XPO1 would augment the clinical translation of XPO1 inhibitors for the treatment of gastric cancer. Nonetheless, the enhanced efficacy and low toxicity profiles of KPT-330 in combination with conventional chemotherapy is promising and adds value to a synergistic interaction that could be utilized in the treatment of refractory gastric cancers.

## Electronic supplementary material

Supplementary File

## References

[1] Crochiere ML (2016). A method for quantification of exportin-1 (XPO1) occupancy by Selective Inhibitor of Nuclear Export (SINE) compounds. Oncotarget.

[2] Turner JG, Sullivan DM (2008). CRM1-mediated nuclear export of proteins and drug resistance in cancer. Curr Med Chem.

[3] Chang, Y. J. *et al*. In *Cell Microbiol* Vol. 8 1740–1752 (2006). 10.1111/j.1462-5822.2006.00743.x 16759223

[4] Xu D, Farmer A, Chook YM (2010). Recognition of nuclear targeting signals by Karyopherin-beta proteins. Curr Opin Struct Biol.

[5] Hill R, Cautain B, de Pedro N, Link W (2014). Targeting nucleocytoplasmic transport in cancer therapy. Oncotarget.

[6] Gravina GL (2014). Nucleo-cytoplasmic transport as a therapeutic target of cancer. J. Hematol Oncol.

[7] Lapalombella R (2012). Selective inhibitors of nuclear export show that CRM1/XPO1 is a target in chronic lymphocytic leukemia. Blood.

[8] Turner JG, Dawson J, Sullivan DM (2012). Nuclear export of proteins and drug resistance in cancer. Biochem Pharmacol.

[9] Zhou F (2013). CRM1 is a novel independent prognostic factor for the poor prognosis of gastric carcinomas. Med Oncol.

[10] Senapedis WT, Baloglu E, Landesman Y (2014). Clinical translation of nuclear export inhibitors in cancer. Semin Cancer Biol.

[11] Newlands ES, Rustin GJ, Brampton MH (1996). Phase I trial of elactocin. Br J Cancer.

[12] Inoue H (2013). CRM1 blockade by selective inhibitors of nuclear export attenuates kidney cancer growth. J Urol.

[13] Azmi AS (2013). Selective inhibitors of nuclear export block pancreatic cancer cell proliferation and reduce tumor growth in mice. Gastroenterology.

[14] Mendonca J (2014). Selective inhibitors of nuclear export (SINE) as novel therapeutics for prostate cancer. Oncotarget.

[15] Soung YH (2017). Selective Inhibitors of Nuclear Export (SINE) compounds block proliferation and migration of triple negative breast cancer cells by restoring expression of ARRDC3. Oncotarget.

[16] Salas Fragomeni RA (2013). CRM1 and BRAF inhibition synergize and induce tumor regression in BRAF-mutant melanoma. Mol Cancer Ther.

[17] Gandhi, U. H. *et al*. Clinical Implications of Targeting XPO1-mediated Nuclear Export in Multiple Myeloma. *Clin Lymphoma Myeloma Leuk*, 10.1016/j.clml.2018.03.003 (2018). 10.1016/j.clml.2018.03.003 29610030

[18] Yoshimura M (2014). Induction of p53-mediated transcription and apoptosis by exportin-1 (XPO1) inhibition in mantle cell lymphoma. Cancer Sci.

[19] Etchin J (2016). Activity of a selective inhibitor of nuclear export, selinexor (KPT-330), against AML-initiating cells engrafted into immunosuppressed NSG mice. Leukemia.

[20] Etchin J (2013). Antileukemic activity of nuclear export inhibitors that spare normal hematopoietic cells. Leukemia.

[21] Muqbil I (2016). Anti-tumor activity of selective inhibitor of nuclear export (SINE) compounds, is enhanced in non-Hodgkin lymphoma through combination with mTOR inhibitor and dexamethasone. Cancer Lett.

[22] Vogl DT (2018). Selective Inhibition of Nuclear Export With Oral Selinexor for Treatment of Relapsed or Refractory Multiple Myeloma. J Clin Oncol.

[23] Ooi CH (2009). Oncogenic pathway combinations predict clinical prognosis in gastric cancer. PLoS Genet.

[24] Turner, J. G. *et al*. XPO1 inhibitor combination therapy with bortezomib or carfilzomib induces nuclear localization of IkappaBalpha and overcomes acquired proteasome inhibitor resistance in human multiple myeloma. *Oncotarget*, 10.18632/oncotarget.12969 (2016). 10.18632/oncotarget.12969 PMC5340237 27806331

[25] Ranganathan P (2016). XPO1 Inhibition using Selinexor Synergizes with Chemotherapy in Acute Myeloid Leukemia by Targeting DNA Repair and Restoring Topoisomerase IIalpha to the Nucleus. Clin Cancer Res.

[26] Etchin J (2013). KPT-330 inhibitor of CRM1 (XPO1)-mediated nuclear export has selective anti-leukaemic activity in preclinical models of T-cell acute lymphoblastic leukaemia and acute myeloid leukaemia. Br J Haematol.

[27] Zheng Y (2014). KPT-330 inhibitor of XPO1-mediated nuclear export has anti-proliferative activity in hepatocellular carcinoma. Cancer Chemother Pharmacol.

[28] Abdul Razak AR (2016). First-in-Class, First-in-Human Phase I Study of Selinexor, a Selective Inhibitor of Nuclear Export, in Patients With Advanced Solid Tumors. J Clin Oncol.

[29] Walker CJ (2013). Preclinical and clinical efficacy of XPO1/CRM1 inhibition by the karyopherin inhibitor KPT-330 in Ph + leukemias. Blood.

[30] Moll UM, Petrenko O (2003). The MDM2-p53 interaction. Mol Cancer Res.

[31] Mutka SC (2009). Identification of nuclear export inhibitors with potent anticancer activity *in vivo*. Cancer Res.

[32] Cmielova J, Rezacova M (2011). p21Cip1/Waf1 protein and its function based on a subcellular localization [corrected]. J Cell Biochem.

[33] Wu, T. *et al*. Nuclear export of ubiquitinated proteins determines the sensitivity of colorectal cancer to proteasome inhibitor. *Mol Cancer Ther*, 10.1158/1535-7163.mct-16-0553 (2016). 10.1158/1535-7163.MCT-16-0553 27903750

[34] Knauer SK, Bier C, Habtemichael N, Stauber RH (2006). The Survivin-Crm1 interaction is essential for chromosomal passenger complex localization and function. EMBO Rep.

[35] Okada E (2001). Survivin expression in tumor cell nuclei is predictive of a favorable prognosis in gastric cancer patients. Cancer Lett.

[36] Li C (2012). Clinicopathological and prognostic significance of survivin over-expression in patients with esophageal squamous cell carcinoma: a meta-analysis. PLoS One.

[37] Altieri DC (2003). Validating survivin as a cancer therapeutic target. Nat Rev Cancer.

[38] Turner JG, Dawson J, Cubitt CL, Baz R, Sullivan DM (2014). Inhibition of CRM1-dependent nuclear export sensitizes malignant cells to cytotoxic and targeted agents. Semin Cancer Biol.

[39] Hye Won Chung, R. A. S. F., Sharon Shacham, Michael Kauffman, James C. Cusack. In *2013 Gastrointestinal Cancers Symposium* Vol. 31 (Journal of clinical oncology, 2013).

[40] Gilbert DC, Chalmers AJ, El-Khamisy SF (2012). Topoisomerase I inhibition in colorectal cancer: biomarkers and therapeutic targets. Br J Cancer.

