# Supplementary material for: Anti-tumor efficacy of Selinexor (KPT-330) in gastric cancer is dependent on nuclear accumulation of p53 tumor suppressor
Source: Sci Rep. 2018 Aug 16;8:12248. doi: 10.1038/s41598-018-30686-1 (PMC6095850; doi:10.1038/s41598-018-30686-1)
Supplement: Supplementary file 1 — Supplementary File [file 41598_2018_30686_MOESM1_ESM.docx]

**Anti-tumor efficacy of Selinixor (KPT-330) in gastric cancer is dependent on nuclear accumulation of p53 tumor suppressor.**

*Vinod Vijay Subhash; Mei Shi Yeo; Lingzhi Wang; Shi Hui Tan; Foong Ying Wong; Win Lwin Thuya; Woei Loon Tan; Praveen C Peethala; Mu Yar Soe; David SP Tan; Nisha Padmanabhan; Erkan Baloglu; Sharon Shacham; Patrick Tan; H. Phillip Koeffler; Wei Peng Yong*

**Supplementary file.**

|  |  |  |  |  |
| --- | --- | --- | --- | --- |
|  | N=153 | XPO1 High  (≥4281.84) | XPO1 Low (<4281.84) | P value (Chi-square) |
|  |  |  |  |  |
|  |  |  |  |  |
|  |  |  |  |  |
|  |  |  |  |  |
|  |  |  |  |  |
| T stage |  |  |  |  |
| T1-T2 | 24 | 12(50%) | 12(50%) | 0.999 |
| T3-T4 | 127 | 64(50.4%) | 63(49.6%) |  |
| Not reported | 2 | 1(50%) | 1(50%) |  |
|  |  |  |  |  |
|  |  |  |  |  |
| Nstage |  |  |  |  |
| N0-N1 | 69 | 31(41.9%) | 38(55.1%) | 0.476 |
| N2-N3 | 82 | 45(54.9%) | 37(45.1%) |  |
| Not reported | 2 | 1(50%) | 1(50%) |  |
|  |  |  |  |  |
| M stage |  |  |  |  |
| M0 | 122 | 64(52.5%) | 58(47.5%) | 0.288 |
| M1 | 30 | 12(40%) | 18(60%) |  |
| Not reported | 1 | 1(100%) | 0(0%) |  |
|  |  |  |  |  |
| Stage (AJCC7) |  |  |  |  |
| Stage1&Stage 2 | 44 | 18(40.9%) | 26(59.1%) | 0.219 |
| Stage 3&Stage 4 | 108 | 58(53.7%) | 50(46.3%) |  |
| Not reported | 1 | 1(100%) | 0(0%) |  |
|  |  |  |  |  |
|  |  |  |  |  |
| Histo Grade |  |  |  |  |
| Well& Mod differentiated | 54 | 29(53.7%) | 25(46.3%) | 0.537 |
| Poorly& Undifferentiated | 99 | 48(48.5%) | 51(51.5%) |  |
|  |  |  |  |  |
| Lauren |  |  |  |  |
| Diffuse | 62 | 24(38.7%) | 38(61.3%) | 0.018 |
| Non-diffuse | 91 | 53(58.2%) | 38(41.8%) |  |
|  |  |  |  |  |

**Supplementary Table S1.** Analysis of correlation between XPO1 expression and pathological factors in patients with GC

(a)

*

*

**YCC10**

**
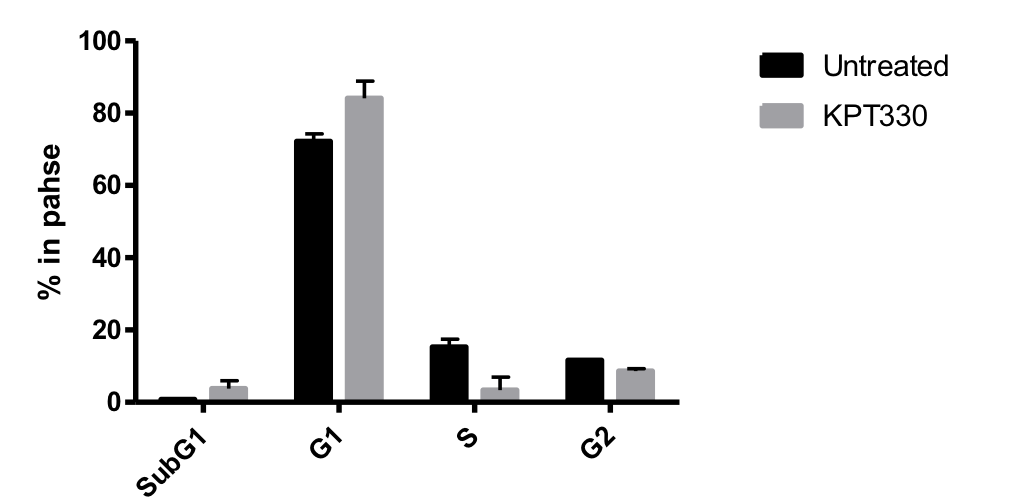
**

(b)

**Untreated**

**KPT-330**

2.2%

0.3%

9.5%

2.4%

**YCC10**

(c)

*

*

**Supplementary Figure S2.** YCC10 cells treated with IC50 concentrations of KPT-330 for 24 hours was analysed for cell cycle profiles and apoptosis (a) Cell cycle analysis (b) Annexin-FITC apoptosis assay. (c) Quantification of apoptosis in KPT-330 treated AGS and YCC10 cells as compared to untreated cells. The results shown are representative of atleast two independent experiments.* *p*-value of less than 0.05 was considered statistically significant

(a)


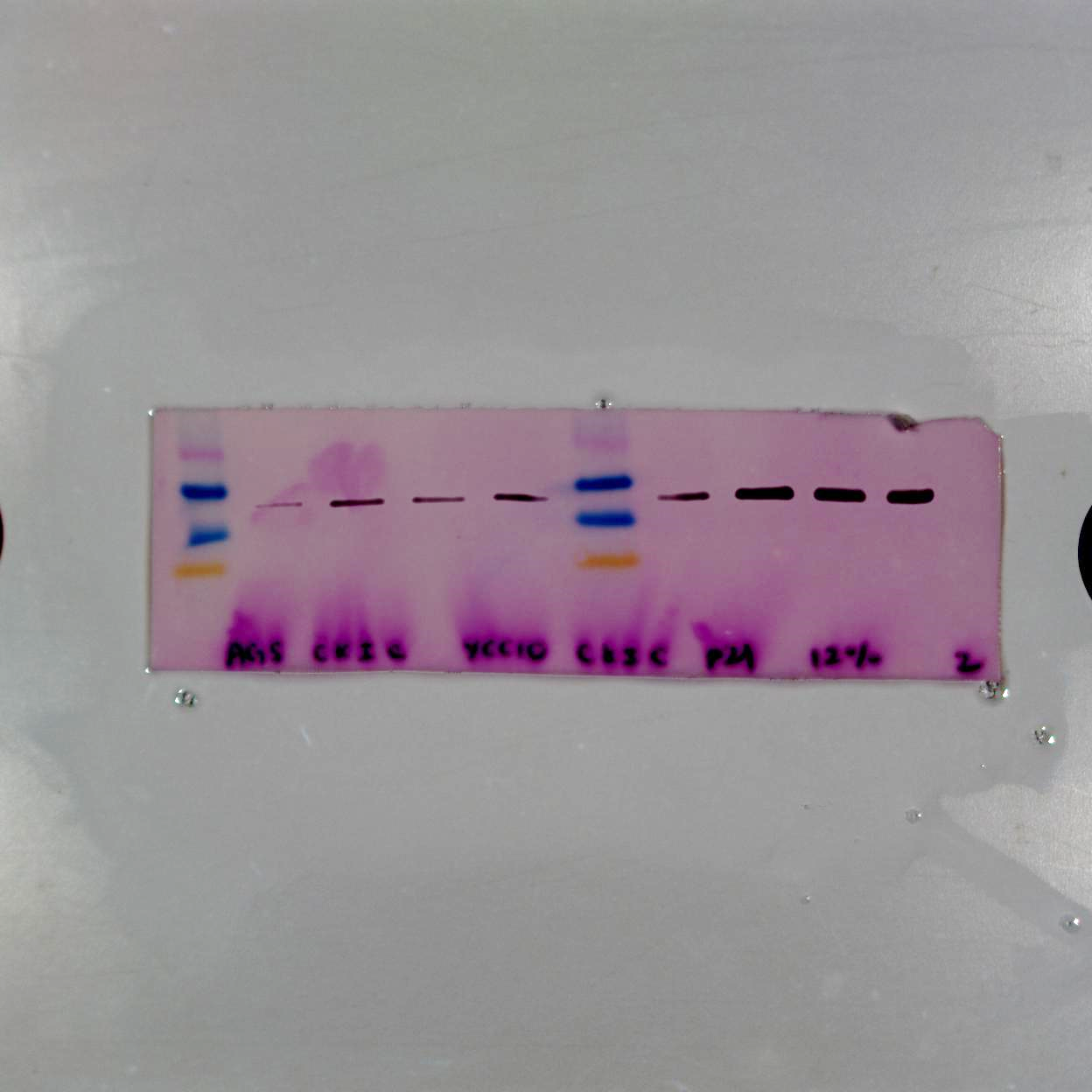

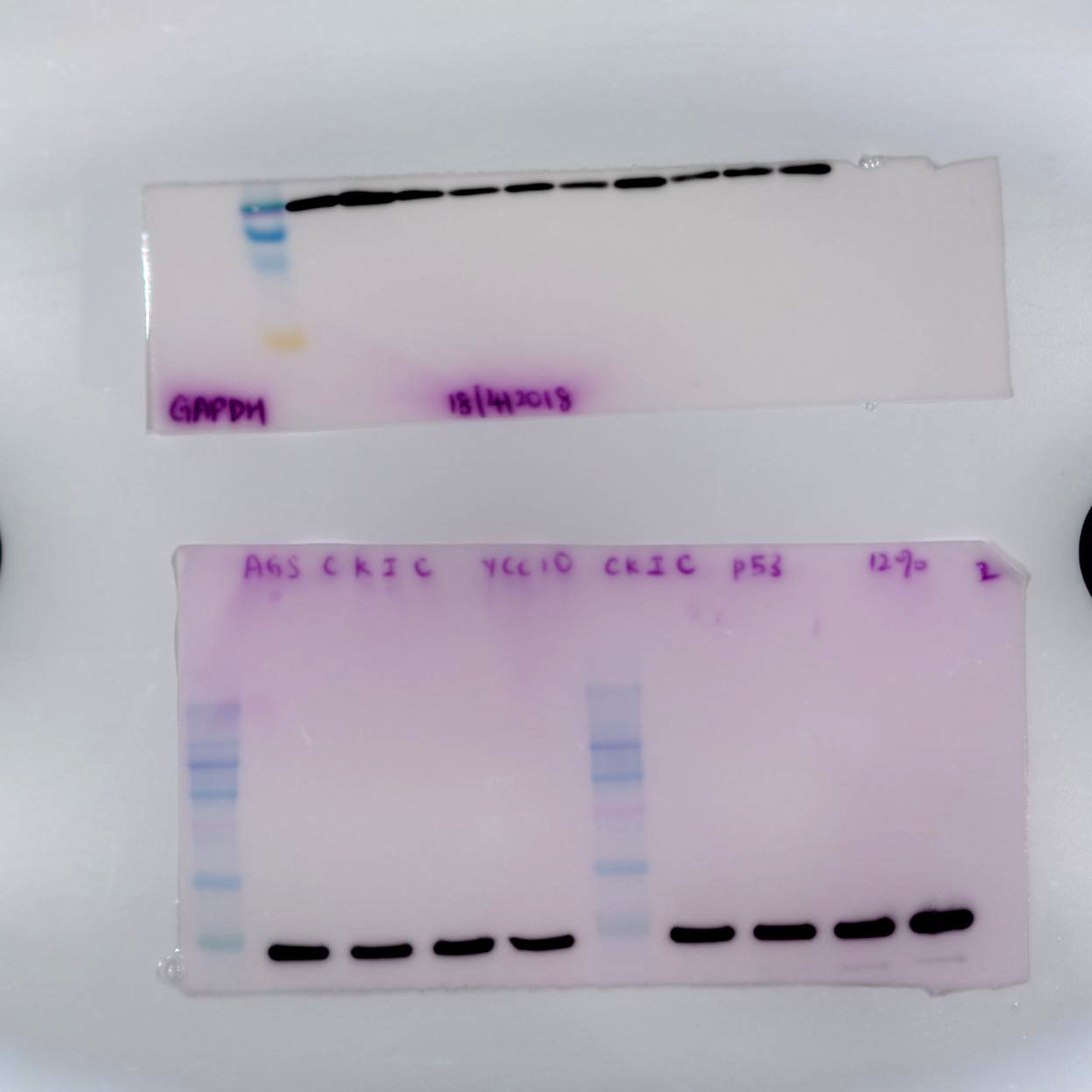

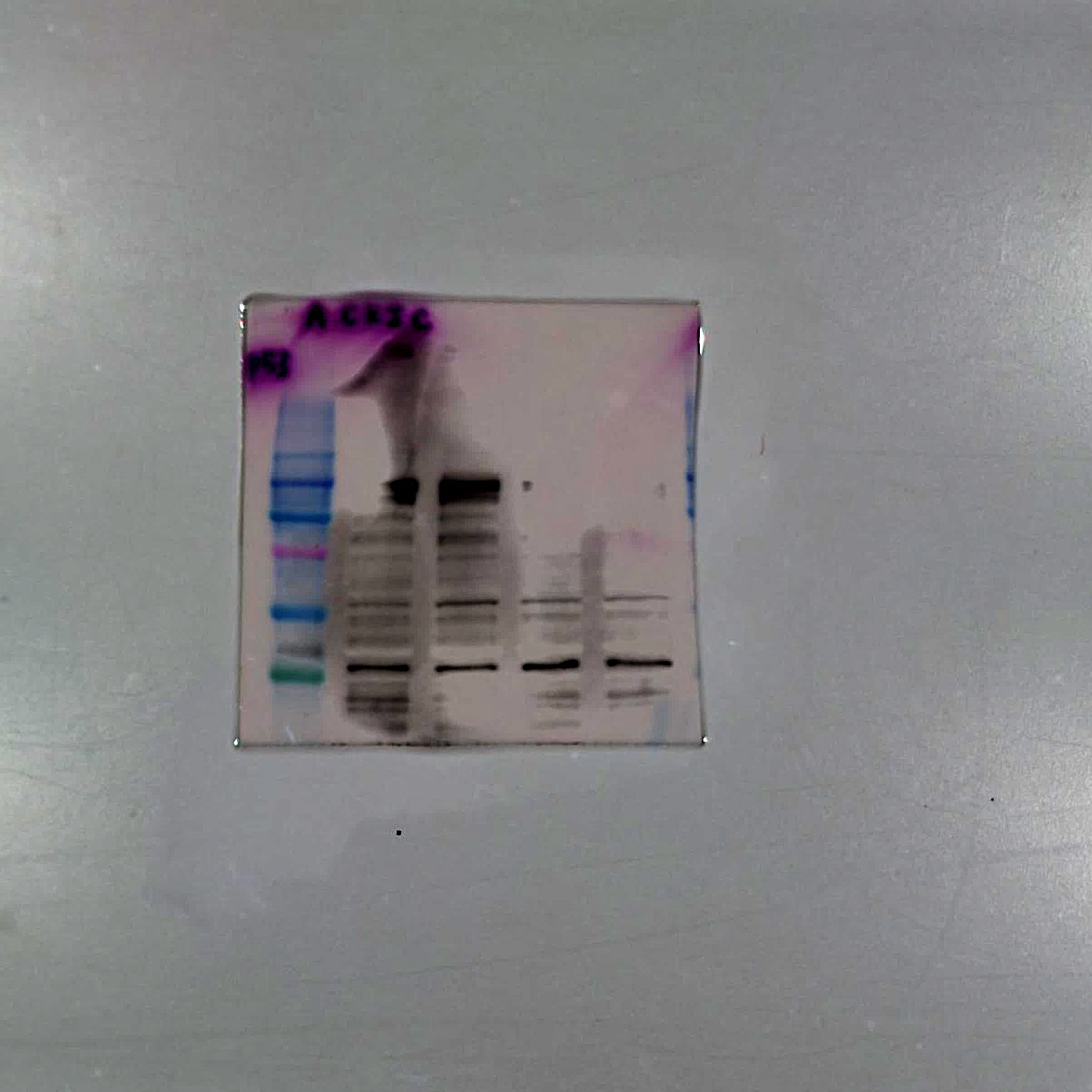


KPT-330 +

Irinotecan

Irinotecan

KPT-330

Ctrl

AGS xenograft

P21

P53

GAPDH

(b)


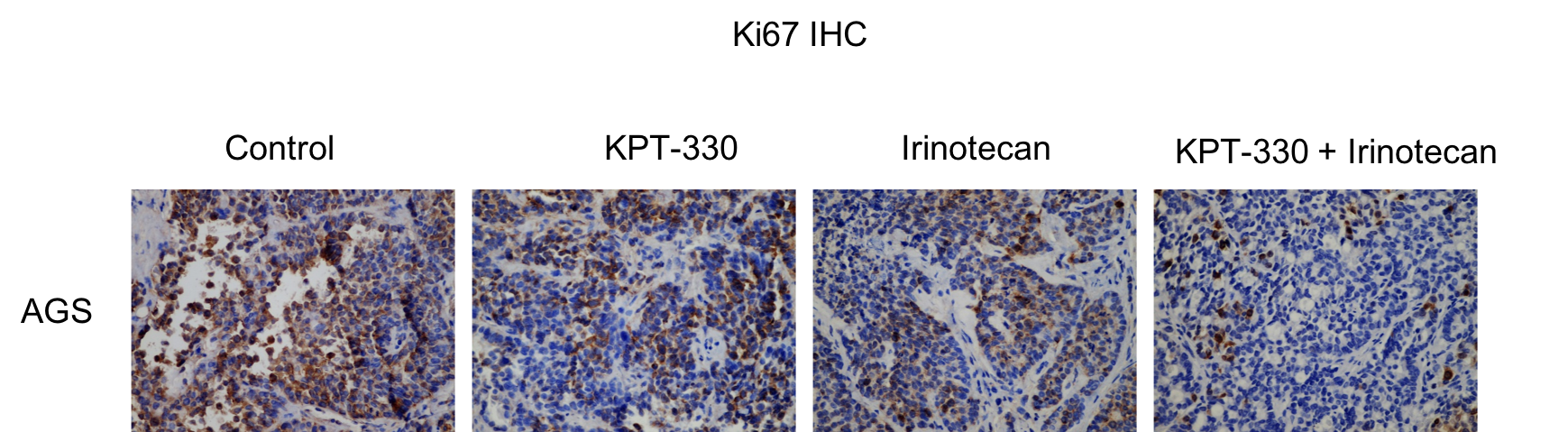


**Supplementary Figure S3. (a)** Immunoblots of p21 and p53 expression in AGS xenograft tissues from NOD-SCID mice treated with KPT-330, Irinotecan, KPT-330 + Irinotecan. GAPDH served as loading control. (b) Immunohistochemical analysis of ki67 expression in AGS xenograft.
